# PROTEOMAS: a workflow enabling harmonized proteomic meta-analysis and proteomic signature mapping

**DOI:** 10.1186/s13321-023-00710-2

**Published:** 2023-03-19

**Authors:** Aileen Bahl, Celine Ibrahim, Kristina Plate, Andrea Haase, Jörn Dengjel, Penny Nymark, Verónica I. Dumit

**Affiliations:** 1 Department of Chemicals and Product Safety, German Federal Institute for Risk Assessment (BfR), Berlin, Germany; 2 University of Fribourg, Fribourg, Switzerland; 3 Institute of Environmental Medicine, Karolinska Institute, Stockholm, Sweden

**Keywords:** Proteomics, Harmonized proteomics data analysis, Meta-analysis, Mode-of-action (MoA), Adverse outcome pathways (AOP), Nanomaterials, FAIR data

## Abstract

Toxicological evaluation of substances in regulation still often relies on animal experiments. Understanding the substances’ mode-of-action is crucial to develop alternative test strategies. Omics methods are promising tools to achieve this goal. Until now, most attention was focused on transcriptomics, while proteomics is not yet routinely applied in toxicology despite the large number of datasets available in public repositories. Exploiting the full potential of these datasets is hampered by differences in measurement procedures and follow-up data processing. Here we present the tool PROTEOMAS, which allows meta-analysis of proteomic data from public origin. The workflow was designed for analyzing proteomic studies in a harmonized way and to ensure transparency in the analysis of proteomic data for regulatory purposes. It agrees with the Omics Reporting Framework guidelines of the OECD with the intention to integrate proteomics to other omic methods in regulatory toxicology. The overarching aim is to contribute to the development of AOPs and to understand the mode of action of substances. To demonstrate the robustness and reliability of our workflow we compared our results to those of the original studies. As a case study, we performed a meta-analysis of 25 proteomic datasets to investigate the toxicological effects of nanomaterials at the lung level. PROTEOMAS is an important contribution to the development of alternative test strategies enabling robust meta-analysis of proteomic data. This workflow commits to the FAIR principles (Findable, Accessible, Interoperable and Reusable) of computational protocols.

**Supplementary Information:**

The online version contains supplementary material available at 10.1186/s13321-023-00710-2.

## Introduction

Animal testing is still key in risk assessment of chemical substances but in vivo experiments imply exorbitant costs. The high number of different toxicological endpoints that need to be evaluated is also a bottleneck when assessing substance toxicity. The increasing number of substances to be introduced in the market calls for the development of reliable alternative methods. The most commonly used experimental alternative models are in vitro tests based on cell cultures that are typically used to assess acute effects. However, to adequately cover more complex endpoints and in particular chronic effects, integrated test strategies that combine a series of different assays are needed. Developing such test strategies requires mechanistic understanding of the underlying biological changes caused by the substances.

In toxicology, a key concept to depict mechanistic knowledge of the effect of a substance at different biological levels is the concept of Adverse Outcome Pathway (AOP), which is a robust framework to contribute to regulatory decision making [1, 2]. AOPs address the alterations induced by a substance at the molecular, cellular, organ and organism level [3] and aim to describe the substance mode-of-action (MoA) [4] as a series of key events. Different in vitro and in silico technologies can then be applied to evaluate the key events preceeding the adverse outcome.

Omics-based technologies became important in toxicology because they allow to investigate toxicity mechanisms in a holistic manner. In this way, they account for the generation of vast datasets at different biological levels [5]. Although these approaches can provide detailed insights into MoA at molecular and cellular levels [6, 7], omics technologies are not yet part of the routine methods in regulatory hazard assessment procedures because standardization of the computational models for interpretation of the datasets is still needed [8, 9]. Workflows for harmonized analysis of omic data contribute directly to facilitate the use of omics in regulatory-decision making.

Among all omic techniques, transcriptomics has an immediate potential in this field, because data generation and analysis can be well harmonized and results allow for straightforward comparison between experiments. However, the major drawback of transcriptomics is the relatively indirect relationship between the measured effects and the respective phenotype. Proteomics, despite being able to describe closer the phenotype, is not generally performed in a harmonized manner. Next to inherent technical challenges, several factors contribute to the lack of uniformity of proteomics measurements: there are no unified experimental design nor sample preparation protocols, and the different degrees of sophistication of the measuring devices result in high level of noise. Additionally, datasets available on public repositories frequently suffer from insufficient metadata, hindering the assignment of the correct experimental condition to each file within the dataset. Moreover, different methods for analyzing and modeling the data often lead to different results, hampering the comparison of the data originated from separate studies. Although analytic methods are equally valid, their pipelines are usually adapted to fit the datasets generating an impact on the outcome. These challenges call for attention if publicly available data is meant to be reused [10].

In this work, we introduce PROTEOMAS, a workflow designed to analyze proteomic studies in a harmonized and transparent manner with the aim to increase their potential for (re)use in toxicological regulatory processes. The workflow follows the Omics Reporting Framework by the Organisation for Economic Cooperation and Development (OECD) [11, 12], precisely to the Data Acquisition and Processing Reporting Module (DAPRM) and to the Data Analysis Reporting Module (DARM) for discovery of differently abundant molecules. It intends to integrate proteomics to transcriptomics and metabolomics, which are so far the only omic techniques further accepted in regulatory matters. During the analysis, a log file collecting all relevant information according to the Omics Reporting Framework is created which guarantees transparency of all steps and results. The overarching aim of PROTEOMAS is to contribute to the understanding of the MoA of substances and to the development of AOPs. Notably, our workflow complies with the FAIR principles (Findable, Accessible, Interoperable and Reusable) of bioinformatics tools, and contributes to data FAIRness of proteomics studies [13].

## Results

The PRIDE Archive, one of the main public repositories for proteomic data, currently hosts over 20,000 projects. This large amount of data has great potential in toxicology. However, it is difficult to use these datasets to compare the outcomes of different projects due to their heterogenic nature. Apart from the technical differences, the large variety of analytic workflows and interpretation tools hinders comparability of the results. The tool that we introduce in this work, PROTEOMAS (PROTEOmics Meta-AnalysiS), can perform automated and harmonized meta-analyses of data-dependent acquisition (DDA) proteomic datasets using the popular and commonly used label-free quantification (LFQ) algorithm of the freeware MaxQuant [14]. PROTEOMAS functions independently of technical specifications and of metadata availability. PROTEOMAS can process results obtained from different devices, with the only condition that generated files can be analyzed with MaxQuant. Currently supported file formats are *.wiff (ABSciex), *.mzxml (MzXml), *.raw (Thermo), *.uimf (UIMF), and *.d (Agilent and Bruker). This approach then enables the comparison of results from different studies.

**Fig. 1:**
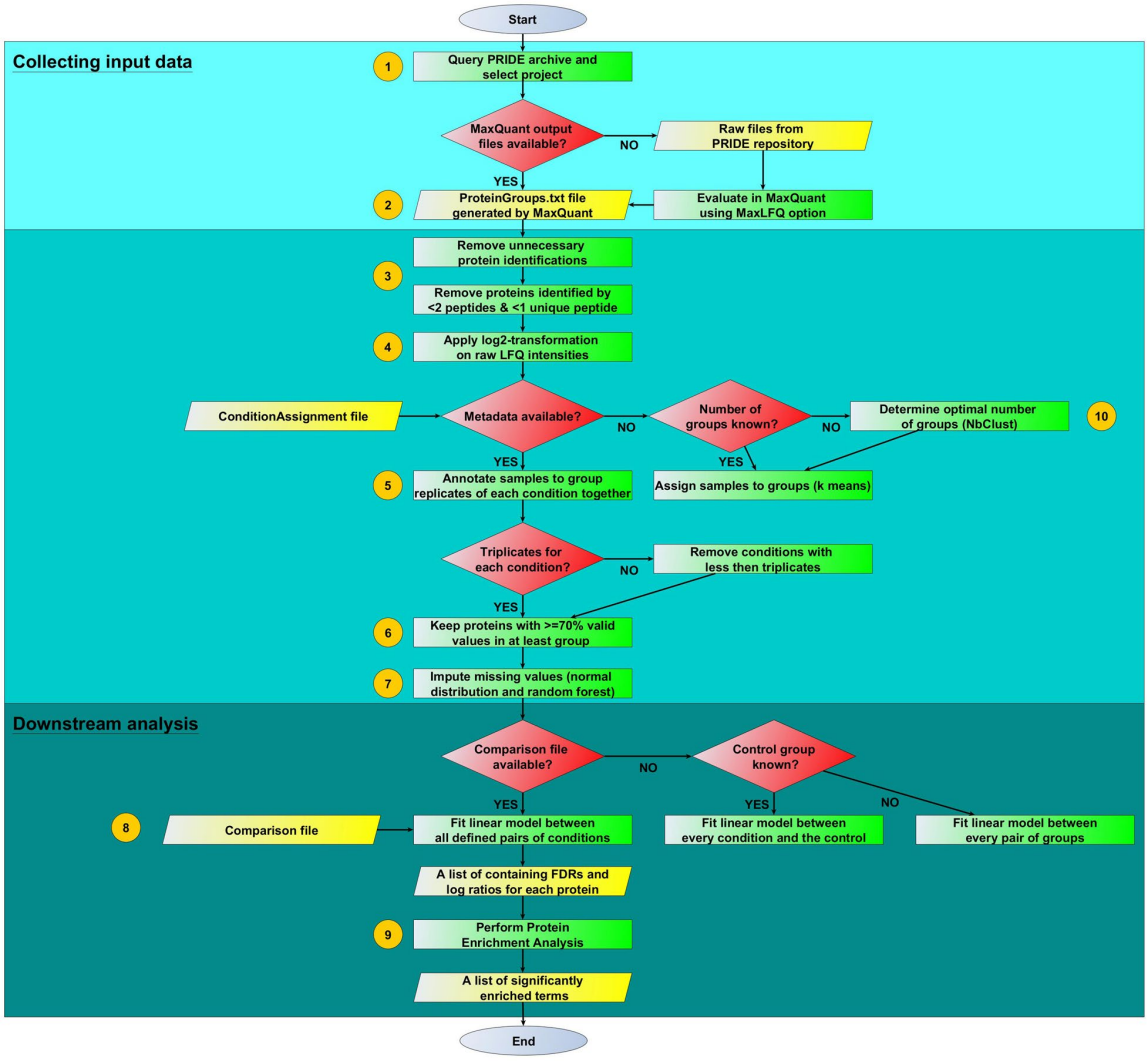
Flowchart showing the processing steps within the workflow. Each implemented step is represented by a separate rectangle (green). Decisions based on certain criteria are represented by diamond shapes (red), while input and output files are shown in parallelograms (yellow). Numbers correspond to the processing steps performed and are further explained in the text

Figure 1 provides an overview on the different processing steps included in the workflow. The **numbers** in the flowchart indicate the different steps which are discussed in detail below. Some steps are decision-based and depend on different criteria regarding the characteristics of the respective datasets and their associated metadata. The workflow can be applied to publicly available datasets from repositories or to newly generated ones.

### Preparation of the proteomic datasets

PROTEOMAS can be used for any dataset generated by a label-free proteomics approach (Fig. 1, step 1). The *sine qua non* condition for the dataset is the possibility to run MaxQuant on it. MaxQuant [14] is a widely used proteomics software for identification and quantification of proteins analyzed by mass spectrometry (MS). Raw files downloaded from the PRIDE repository have to be analyzed with MaxQuant including the option 'LFQ intensities' before running PROTEOMAS. This setting allows for a generic normalization and quantification technique called MaxLFQ [15]. MaxLFQ performs delayed normalization in combination with maximum peptide ratio extraction. Thereby, it solves two common problems occurring during quantification of label-free proteomics data: (1) Delayed normalization removes biases occurring from slight differences in handling and MS performance between sample fractions. The only assumption here is that most proteins do only change minimally between experimental conditions. (2) The maximum peptide ratio extraction algorithm defines the selection of peptide signals which contribute to the overall protein signal across samples. It calculates all pairwise protein ratios among samples based on all shared peptides belonging to the protein of interest. By default, at least two peptide ratios are needed to obtain a valid protein ratio. This default value was not changed in the analysis. As a last step, LFQ intensity profiles are calculated for each protein such that all pairwise peptide comparisons are satisfied and the best estimate is obtained. The underlying assumption of MaxLFQ is that the majority of proteins is not changing between analyzed conditions. However, in the original publication of MaxLFQ, the authors tested this assumption in a benchmark dataset in which more than 30% of all identified proteins were changed. While there was a shift in total log ratios between changed and non-changed proteins, changed proteins could still be detected and quantified as such [15].

MaxQuant generates, among others, a 'proteinGroups.txt' file as output, which is the main input required for running PROTEOMAS (Fig. 1, step 2). In addition to the ‘proteinGroups.txt’ file, the user can create three additional optional files, where information about each measured sample can be described: (1) The ‘ConditionAssignment.csv’ should be used if each treatment condition is known for each sample. Within this file each original sample name is assigned to the associated condition. (2) A more precise specification of the comparisons to be evaluated (e.g. treatment 1 *vs*. control 1 and treatment 2 *vs*. control 2) can be transferred to the workflow through the next optional file called 'Comparisons.csv'. (3) If, on the other hand, information about the treatment conditions is unknown, the 'ClusterNumber.csv' file may be used if the total number of different treatments conditions is known.

### Data pre-processing

Within PROTEOMAS data pre-processing starts by loading the ‘proteinGroups.txt’ file. The input file is pre-processed to remove non-relevant data (Fig. 1, step 3), precisely, proteins marked as ‘contaminants’, ‘identified only by site’ or ‘reverse’. To minimize protein misidentification, only proteins identified by at least two peptides with at least one of them being unique were kept for downstream analysis. In addition, data was log2-transformed (Fig. 1, step 4). In a technically sound dataset, one would expect that log2-transformed values of the LFQ intensities show a normal distribution when plotted as histograms, which in the next steps supports the use of downstream analysis methods which often assume normally distributed data. The workflow produces the corresponding figures and collects them directly in the output folder. This information can be used for analyzing data quality. No external data normalization is performed as this step is inherently done with MaxQuant using the MaxLFQ option.

DDA proteomic datasets typically contain a large number of missing values, which are listed as zeros in the output files. A missing value in the dataset does not necessarily mean that the respective protein was not present in the sample; it means that there were too few data points for proper quantification [16]. After a log2-transformation, values equal to zero will be converted to non-assigned numbers (NaN). In case sufficient metadata is available, all samples will be assigned to their corresponding condition as indicated in the 'ConditionAssignment.csv' file before continuing the analysis (Fig. 1, step 5). The case of missing metadata is described later on. After assigning samples to their respective conditions, it is checked whether at least triplicates are present in each group as this is a minimal pre-requisite for successful outlier detection and statistical testing. If this is not the case for some of the conditions, those are deleted from the dataset.

### Dealing with missing values

A threshold of minimal valid values for each protein entry of 70% in at least one condition group was set as default (Fig. 1, step 6). After filtering proteins, some missing values will very likely remain in the datasets. These values can be replaced by valid values by a process called imputation (Fig. 1, step 7). Imputation allows to retain the full sample size of detected proteins [17], which can contribute to improving the proteome coverage and the determination of enriched descriptors.

There are different imputation methods, which can be divided into two classes: MCAR and MNAR methods [17, 18]. In MCAR methods, values are assumed to be missing completely at random. In the case of proteomics, this would mean that only by chance peptides were not detected by the mass spectrometer. As an example, this could happen if a more abundant peptide elutes at the same time and overshadows the presence of another peptide, which goes undetected. In that case, missing values would optimally be replaced by values, which are in the same range as those of the other replicates within this condition for the protein under consideration. Available methods comprise, e.g. the k-nearest neighbors (kNN) method [19] or the random forest (RF) method [20]. In contrast, MNAR methods assume that values are missing not at random and thus the protein is truly absent. Common examples of MNAR imputation methods are replacement by LOD (limit of detection) values or sampling from a downshifted and shrunk normal distribution which means that missing values are replaced by small values. In proteomic datasets, one would usually expect to see both, MCAR and MNAR values; however, it is impossible to determine the exact type for each missing value. Therefore, within the workflow, we used a combination of MCAR and MNAR methods and the decision on which one to use is based on the amount of missing values across samples within a condition.

**Fig. 2:**
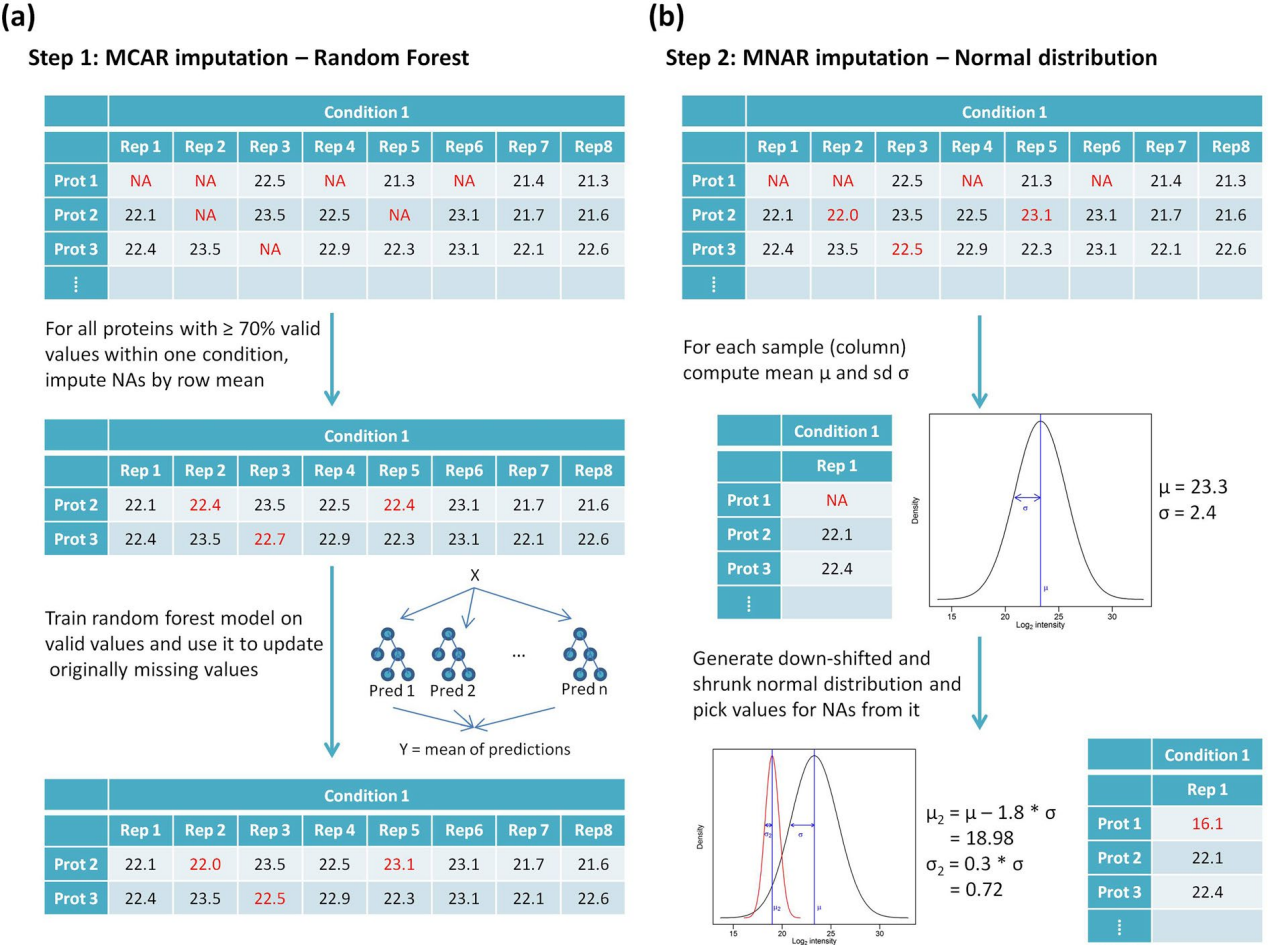
MCAR *vs*. MNAR imputation. Within the PROTEOMAS workflow these are implemented in terms of **a** Step 1: random forest imputation and **b** Step 2: imputation from a down-shifted and shrunk normal distribution

First, we extract all proteins which have at most 30% missing values within the specified condition. We assume that these are actually MCAR values as they were detected in most replicates of the same condition. For those, missing values are replaced with random forest imputation as in that case, we would actually expect the protein to be present in all samples of that condition (in concordance with the filtering based on valid values) (Fig. 2). Random forest imputation starts with replacing all missing values by the mean value of that protein within a given condition and then generating random forest models each time leaving out one of the originally missing values. Each random forest model then predicts a new value which replaces the mean value. This step is done iteratively in order to obtain better results.

**Fig. 3:**
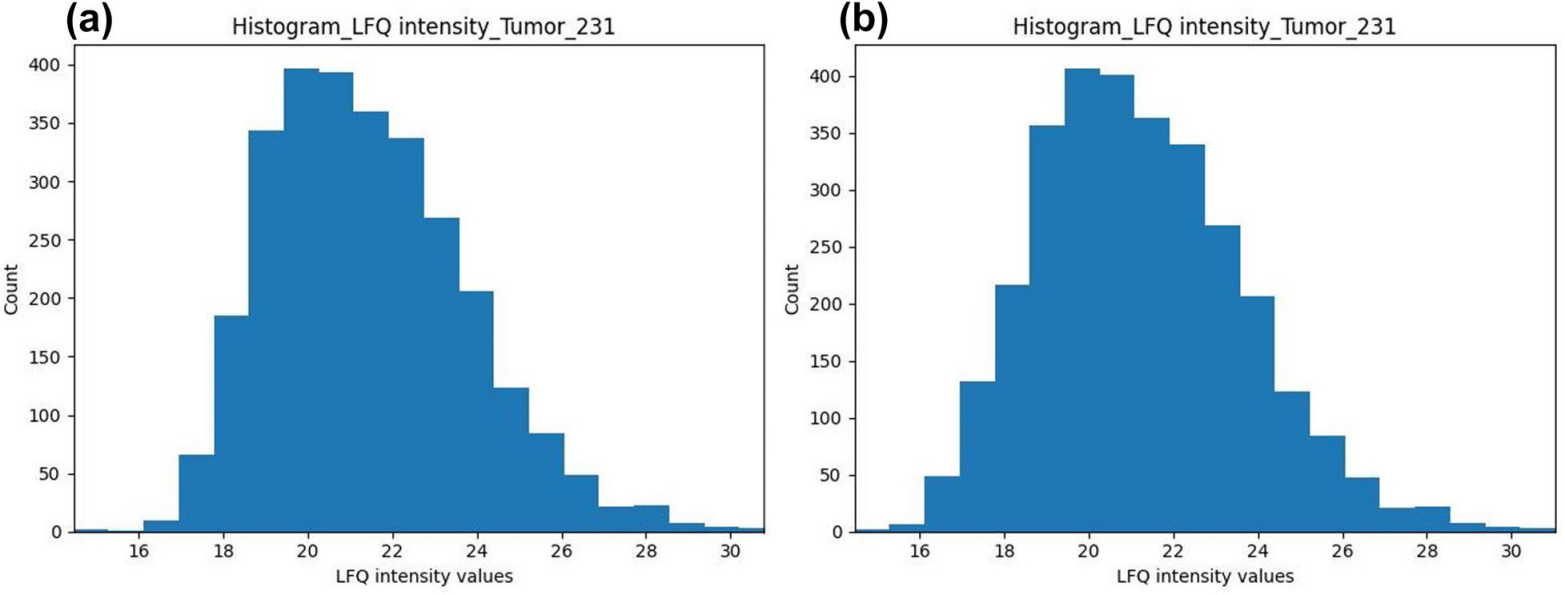
Histograms comparing the data distribution **a** before and **b** after imputation

**Fig. 4:**
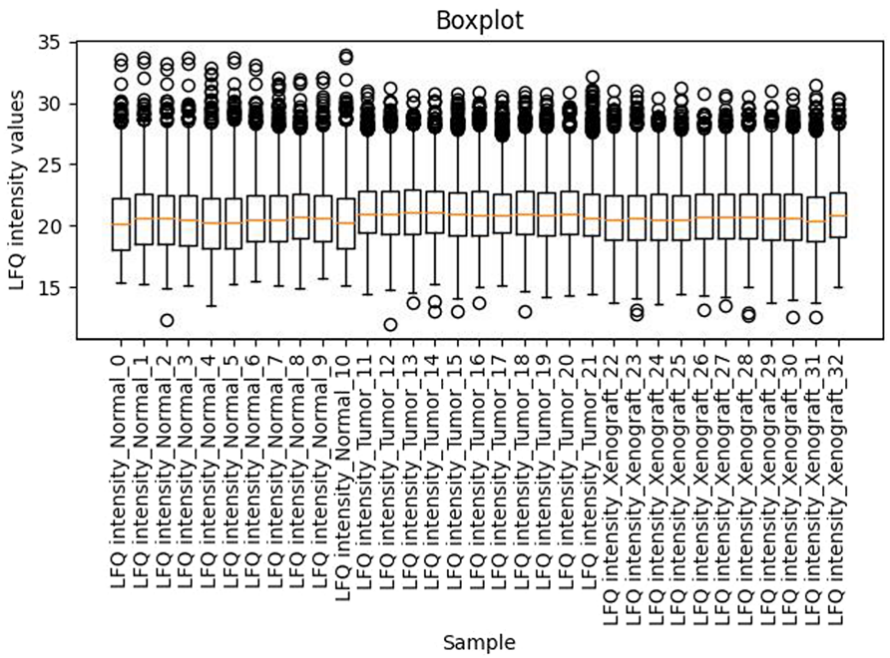
Boxplot showing the comparability of samples after MaxLFQ normalization

After random forest imputation, all other missing values are assumed to be MNAR values and are thus replaced by small values obtained from an imputation based on drawing values from the downshifted and shrunk normal distribution (Fig. 3). In this approach, the width and the center of each sample are calculated separately to simulate random values, which are used to fill the missing values of each sample, such that the width of the distribution will shrink to a factor of 0.3 (default) and the distribution will be downshifted by 1.8 (default) standard deviations (sampling from the left side of the distribution) for each sample. Histograms can be used to check the imputation and dataset quality. An example of histograms before and after imputation is shown in Fig. 3. In addition, Fig. 4 shows a boxplot confirming that samples are comparable and no further normalization is needed after MaxLFQ. The complete collection of quality control plots can be found in the GitHub repository.

### Differential analysis of quantitative changes in protein levels

**Fig. 5:**
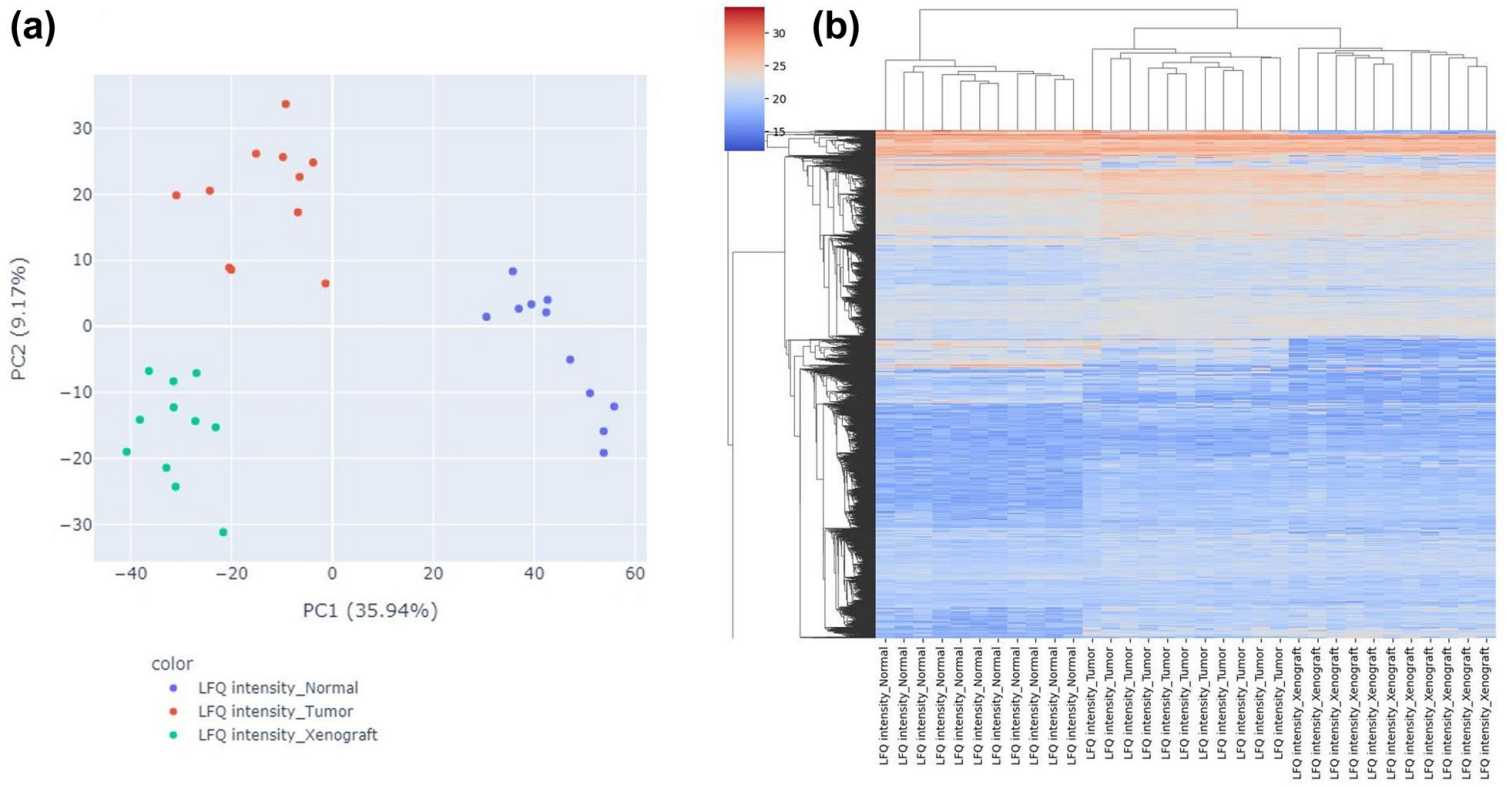
**a** PCA plot and **b** heatmap showing clustering of samples within the different conditions of project ‘PXD000853’

**Fig. 6:**
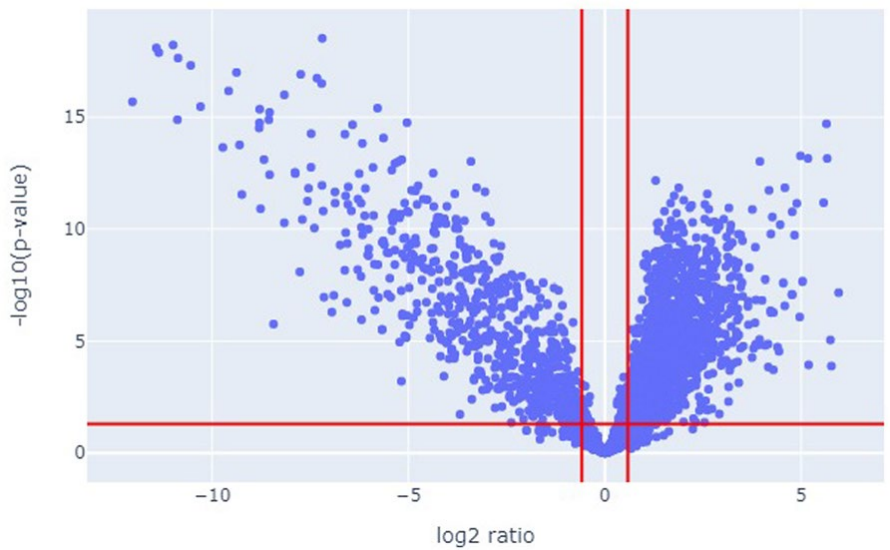
Volcano plot showing differentially abundant proteins between healthy humans and mice with xenograft tumors for project ‘PXD000853’

Remaining proteins including both valid and imputed values are subjected to differential analysis. This allows to detect changes in protein levels between different samples or conditions, while determining along the degree of statistical significance. Here, we used linear modeling (Fig. 1, step 8) to identify proteins which show a significant difference in abundance between two conditions. A Benjamini–Hochberg FDR threshold of 0.05 is used to correct for multiple testing. A fold change threshold of 1.5 up- or downregulation was set for determining significantly changed protein levels between two conditions. For visual inspection of the results, the workflow creates PCA plots and heatmap (Fig. 5) showing the clustering of all groups within one project as well as volcano plots for each comparison (Fig. 6). Plots for all projects are provided in the GitHub repository.

The conditions to be tested against each other can be defined in the 'Condition.csv' file. If no such file is specified, PROTEOMAS will look for any condition named 'control' and compares all conditions against this one. In case no 'Condition.csv' file, as well as no 'control' condition is available, all pairs of conditions are compared against each other.

### Dealing with (missing) metadata

As it is often the case in repositories, the lack of metadata adjoining the datasets hampers proper comparison among treatments or conditions, as the relationship between raw files and corresponding measured samples is not clear. It is still possible to identify clusters of samples according to similarities of protein patterns, but typically criteria to separate treatment or condition groups remains subjective. An additional difficulty arises if the number of conditions evaluated in the dataset is unknown. PROTEOMAS, on the other hand, is able to perform assignment of conditions to each sample in an automated and objective fashion, without subjective bias.

In case metadata is not sufficient to directly assign experimental conditions to each sample, an additional automated condition assignment step is included in PROTEOMAS (Fig. 1, step 10). Here, each sample will be assigned to its condition group using a k-means clustering approach. In k-means clustering, k random cluster centers are defined and each sample is assigned to its nearest cluster center based on Euclidean distance. Then cluster centers are recalculated based on the assigned samples and samples are reassigned to the new center means. This is continued in an iterative fashion until the algorithm converges and group assignments no longer change.

The crucial point in k-means clustering is the value of k, which is the number of clusters to define. In case group assignments cannot directly be obtained from the metadata but still the number of groups is known, k-means algorithm can be performed directly. Otherwise, if the number of groups is also unknown, k first has to be determined. Although the determination of the optimal number of groups could be done by visual inspection of hierarchical clustering or PCA plots, this option is not feasible when processing a large number of projects, and it implies a subjective bias. Therefore, the determination of the optimal number of clusters k is done automatically in this workflow. Multiple methods for detecting the optimal number of k exist and a number of them are implemented in the R-package 'NbClust' [21].

**Fig. 7:**
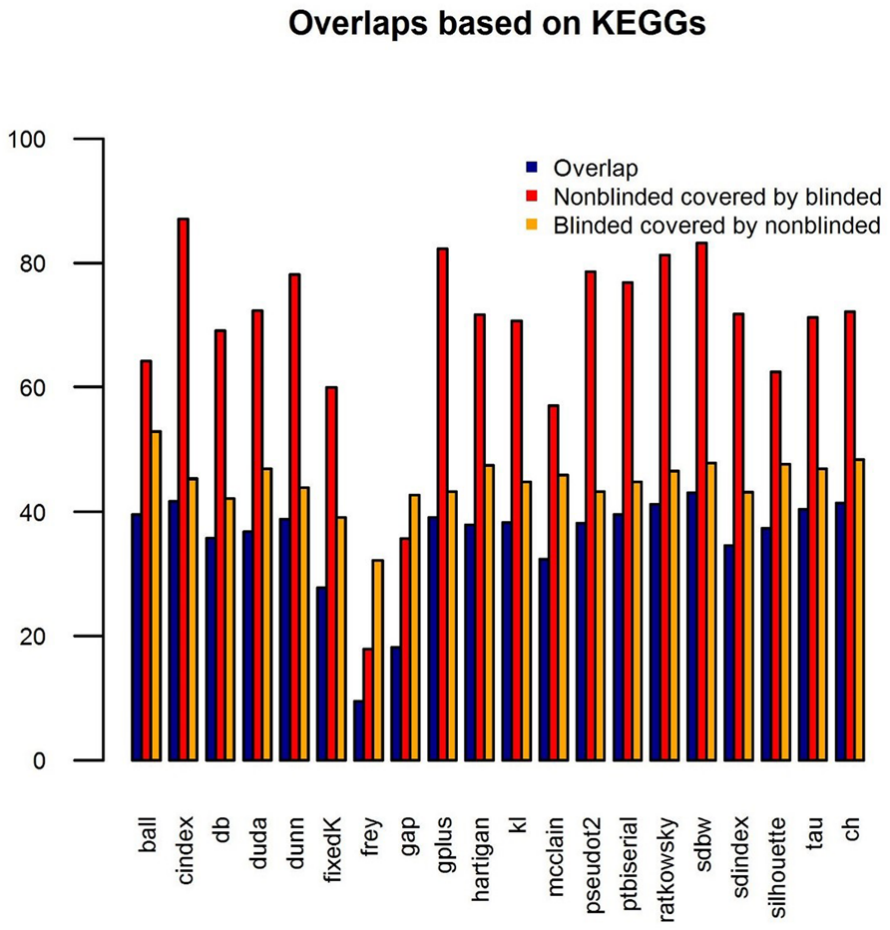
Barplot showing the percentage of overlapping identified KEGG pathways between blinded and nonblinded studies for different indices used to determine the optimal number of clusters k. Each bar represents the mean percentage across all analyzed projects. For each index, the overlap between nonblinded and blinded is shown in blue, the percentage of KEGG pathways in the nonblinded dataset that could also be determined with the blinded setting is shown in red and the percentage of KEGG pathways in the blinded setting that was also present in the original nonblinded setting is depicted in yellow

**Fig. 8:**
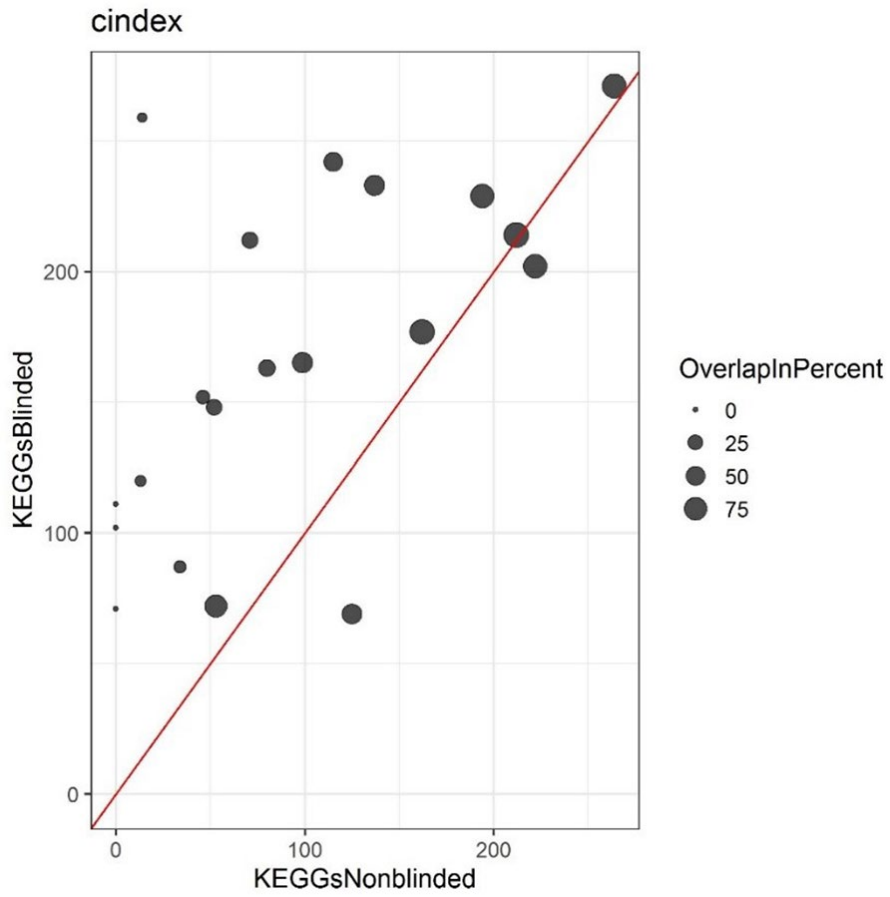
Amount of KEGG pathways found to be significantly altered in the nonblinded, as well as the blinded setting for each analyzed project. Percent overlaps are shown by the size of the dots

**Fig. 9:**
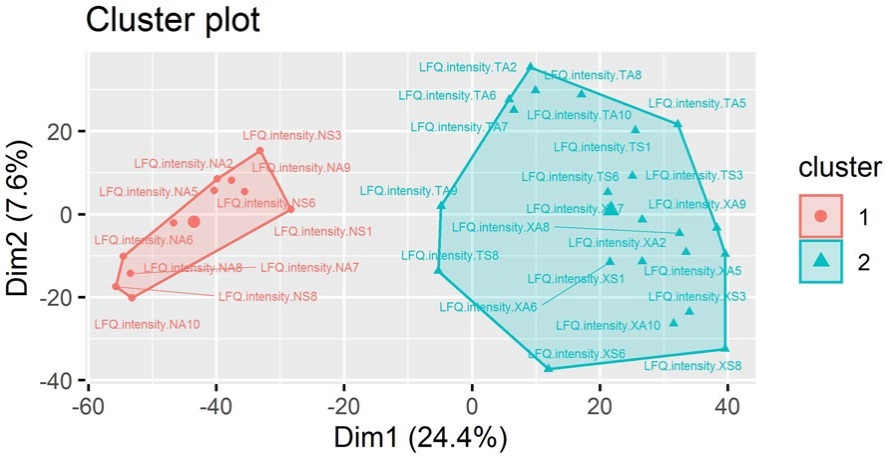
Example of automatic cluster detection (project ‘PXD000853’). The optimal number of clusters k is obtained from method cindex. This choice of k is then used for group assignment using a k-means clustering approach. The cluster plot indicates the group assignment of each sample after k-means clustering with the predicted number of conditions (in this case 2)

To find the most suitable method for determining the optimal k and at the same time also assess the quality of the condition assignment using k-means, we blinded all studies considered for the case study below, which do have sufficient metadata and compared the outcomes in terms of significant proteins (Fig. 7) and KEGG pathways of the blinded and the nonblinded approach (Fig. 8). For the final implementation of PROTEOMAS, cindex was chosen for determining the optimal number of clusters k as it shows the highest recovery of KEGG pathways. Figure 8 shows the amount of KEGG pathways found to be significantly altered in the nonblinded and blinded setting, as well as their overlap for each analyzed project. For blinded analyses, the determined number of k is used as the number of cluster centers to be used for k-means clustering. Each sample of the dataset is then assigned to one of the clusters. An example of the condition assignment by k-means is shown in Fig. 9. Index ‘fixedK' corresponds to the case when the number of conditions is set manually by the user using the ‘ClusterNumber.csv’. Other plots corresponding to this step can be found on the GitHub repository.

### Protein enrichment analysis

Gene set enrichment analysis (GSEA) was used to identify enriched KEGG, Reactome and HALLMARK pathways as well as GO terms (Fig. 1, step 9). For each condition, the list of proteins is sorted in ascending order using the following formula: -log10 (FDR) * abs (log ratio). Thereby, proteins with small FDR and large log ratio are shifted to the beginning of the list, while those with large FDR and small log ratio occur at the end of the list. GSEA then identifies gene set which show over-representation at the top of the protein list. If enough proteins of a gene set are ranked at the top of the list, the gene set will show significant enrichment. These significantly enriched gene sets are collected in the final output file of PROTEOMAS and constitute the proteomic fingerprint of the studied condition.

### Comparison with the original findings

**Fig. 10:**
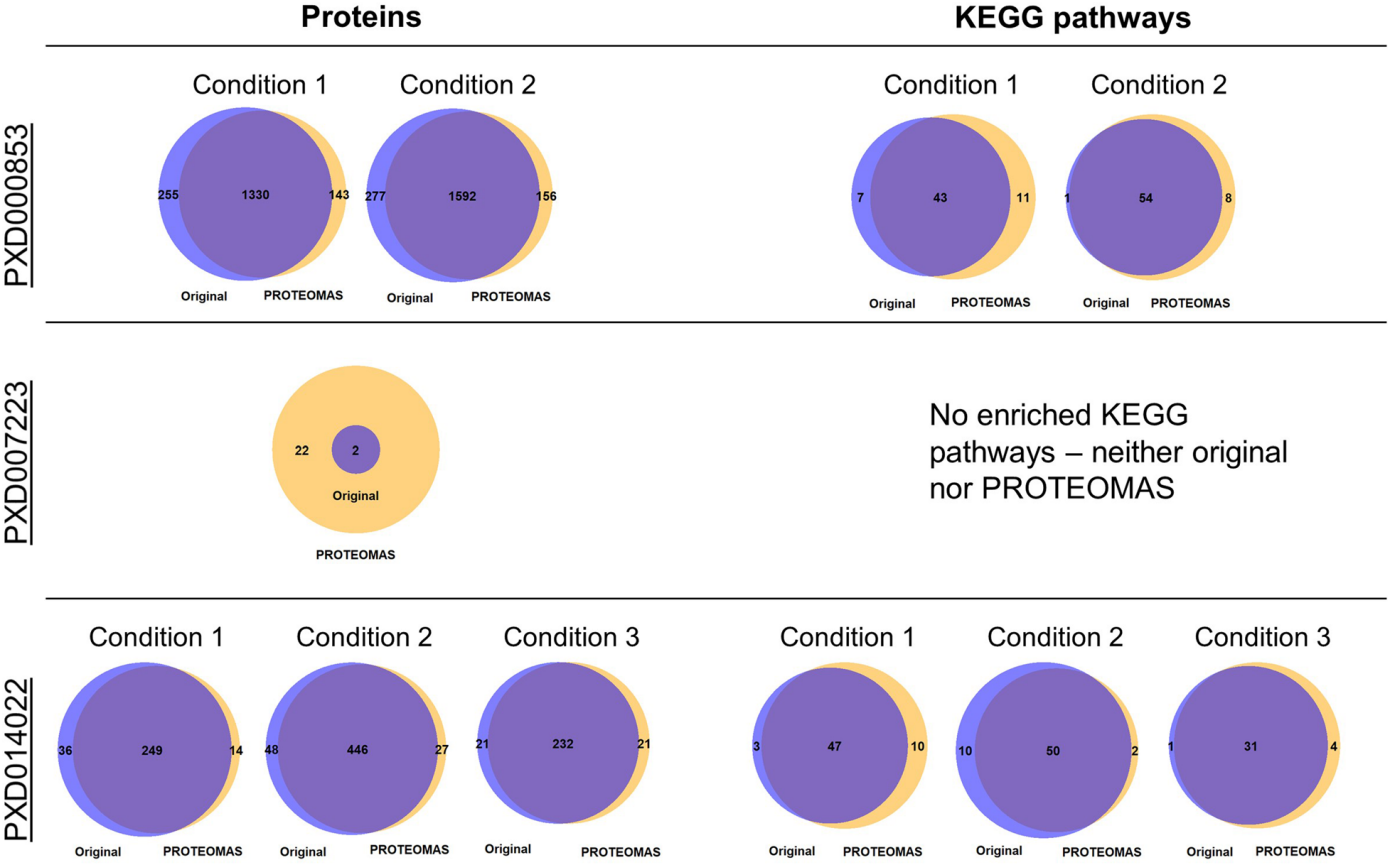
Venn diagrams showing the overlap of the original findings for projects ‘PXD007223’, ‘PXD000853’ and ‘PXD014022’, and those obtained from PROTEOMAS

In order to compare results from the analysis with our workflow to the original ones, we extracted lists of significantly altered proteins from the publications for randomly selected studies [22–24], and performed enrichment analysis of KEGG pathways. Original results were then compared to the lists we obtained using PROTEOMAS. Results of these comparisons are shown in Fig. 10. For all projects under consideration, we see very similar trends: PROTEOMAS consistently finds a similar set of significantly altered proteins and KEGG pathways like the original publications. In all cases, the major part of proteins and pathways are shared between the original publications and PROTEOMAS. Overlaps between original findings and those from our workflow range from 19 to 86% at the protein level and 38% to 85% for KEGG pathways. Therefore, especially on the level of KEGG pathways, which is assumed to be less prone to false-positive findings, PROTEOMAS gives very similar results. In addition, PROTEOMAS detects only very few additional pathways and thus creates only minimal noise.

### Case study: comparison of the proteomic fingerprint of different projects related to lung conditions

As a case study, we tested the workflow to evaluate the toxicological effects of nanomaterials (NMs) at the lung level. NMs consist of particles of which at least 50% are 1 to 100 nm in size in terms of at least one external dimension [25]. Several comprehensive projects have studied the effect of NMs by omics measurements from in vivo and in vitro experiments. Although these results have contributed to the understanding the NMs hazards, the collected information is still not yet sufficient to conclusively unravel different MoA in detail, since the number of NM omic datasets is still comparatively low. However, one may assume that NMs will to a large extent share common MoA with conventional chemicals or other conditions. It is likely that NMs will have unique initiating events, but the following downstream physiological changes are likely to be shared by other effectors. As for NMs inhalation is considered the most critical route of exposure, we kept the focus of our work on lung proteomic datasets. Within this case study, we demonstrate how by means of PROTEOMAS, we are able to extract mechanistic information from different proteomic studies publicly available.

We analyzed 25 lung-related proteomics studies obtained from the PRIDE Archive within this case study. These include studies on lung cancer, pulmonary fibrosis, invasive pulmonary aspergillosis (IPA), chronic obstructive pulmonary disease (COPD), SARS-CoV-2 (Covid-19), and various NM treatments. Table 1 provides information on some project characteristics, as well as the total number of identified proteins, as obtained from the ‘proteinGroups.txt’ file, and it indicates whether metadata to the corresponding dataset is available. Table 2 shows the number of significantly altered proteins as well as enriched descriptors, which constitute the proteomic fingerprint for each comparison.

**Table 1:** Overview of proteomic datasets used in this case study and their characteristics.

Project id	Species	In vitro/ in vivo model	Trait	Total number of proteins in raw data	Sufficient metadata available?	Number of pairs of conditions compared
PXD007223	Human	A549	Lung cancer	2008	Yes	1
PXD000861	Human	BEAS-2B	Lung cancer	3670	Yes	4
PXD018895	Human	A549	Lung cancer	3744	Yes	1
PXD000853	Human	A549	Lung cancer	5197	Yes	2
PXD005698	Human	A549, H358	Lung cancer	942	Yes	2
PXD005733	Human	Lung cancer and adjacent tissue	Lung cancer	1936	Yes	1
PXD007137*	Human	NCI-H650	Lung cancer	1321	No	2
PXD004818*	Human	Lung tissue	Lung cancer	2811	No	6
PXD007180	Human	A549	Smoking	2590	Yes	4
PXD020470	Human	HPA-HULEC co-culture	SARS CoV-2	6753	Yes	2
PXD021685	Human	THP-1	SARS CoV-2	1787	Yes	2
PXD007148h	Human	A549	COPD	466	Yes	2
PXD007148m	Mouse	Lung tissue	COPD	875	Yes	2
PXD016664h	Human	Lung tissue and BALF	IPA	5118	Yes	1
PXD016664m	Mouse	Lung tissue and BALF	IPA	3054	Yes	2
PXD014022	Human	A549	IPA and P. aeruginosa infection	4184		3
PXD005834*	Mouse	A549	IPA	2790	No	4
PXD018569	Human	NCI-H2030	> 30 drugs	8773	Yes	27
PXD023041*	Mouse	Lung tissue	Influenza	3440	No	0
PXD013244	Mouse	Blood serum	Gu-Ben-Fang-Xiao decoction (GBFXD)	3429	Yes	2
PXD016148	Mouse	BALF	NMs (Fe, Co, CB)	1525	Yes	22
PXD019267	Human	THP-1	31 NMs	3665	Yes	33
PXD018900	Rat	BALF	NM-401 (MWCNT)	1223	Yes	8
PXD005970	Human	HBEC-3KT	NM-400 (MWCNT)	5483	Yes	2
PXD025423	Human	HBEC-3KT	NM-62002a (TiO2)	5483	Yes	2

Projects marked with * had insufficient metadata

**Table 2:** Overview on the number of significantly enriched gene set for different background datasets for each analyzed trait in the case study

Trait	# Significantly altered GO terms	# Significantly altered KEGG pathways	# Significantly altered REACTOME pathways	# Significantly altered HALLMARK sets
Lung cancer	1462	46	181	35
IPA	1065	42	189	22
CNT NM-400	396	11	44	12
COPD	312	6	110	8
Drug	1739	47	329	25
CB	19	0	6	0
Co NM	6	1	7	2
Fe NM	80	1	18	0
FeCo NM	127	1	30	0
CNT NM-403	201	2	13	3
Ag NM	185	6	21	5
Au NM	417	10	93	9
Other CNT	300	6	79	8
CuO NM	300	3	65	9
ND	145	1	10	1
LPS	140	4	19	7
QD	276	8	66	8
TiO2	696	33	171	17
Virus	446	21	73	16

### Comparing proteomic signatures across multiple datasets

Evaluated projects usually contain more than one condition, since different treatment, time-points, concentrations, etc., belong to the same dataset. For the present analysis, we have merged the conditions into traits, resulting in the following categories: lung cancer, aspergillosis, COPD, different drug treatments, viral infection, different NMs like carbon, ion releasing and TiO_2_ NMs, among others. Each trait was normalized by the amount of conditions included for an equilibrated comparability.

**Fig. 11:**
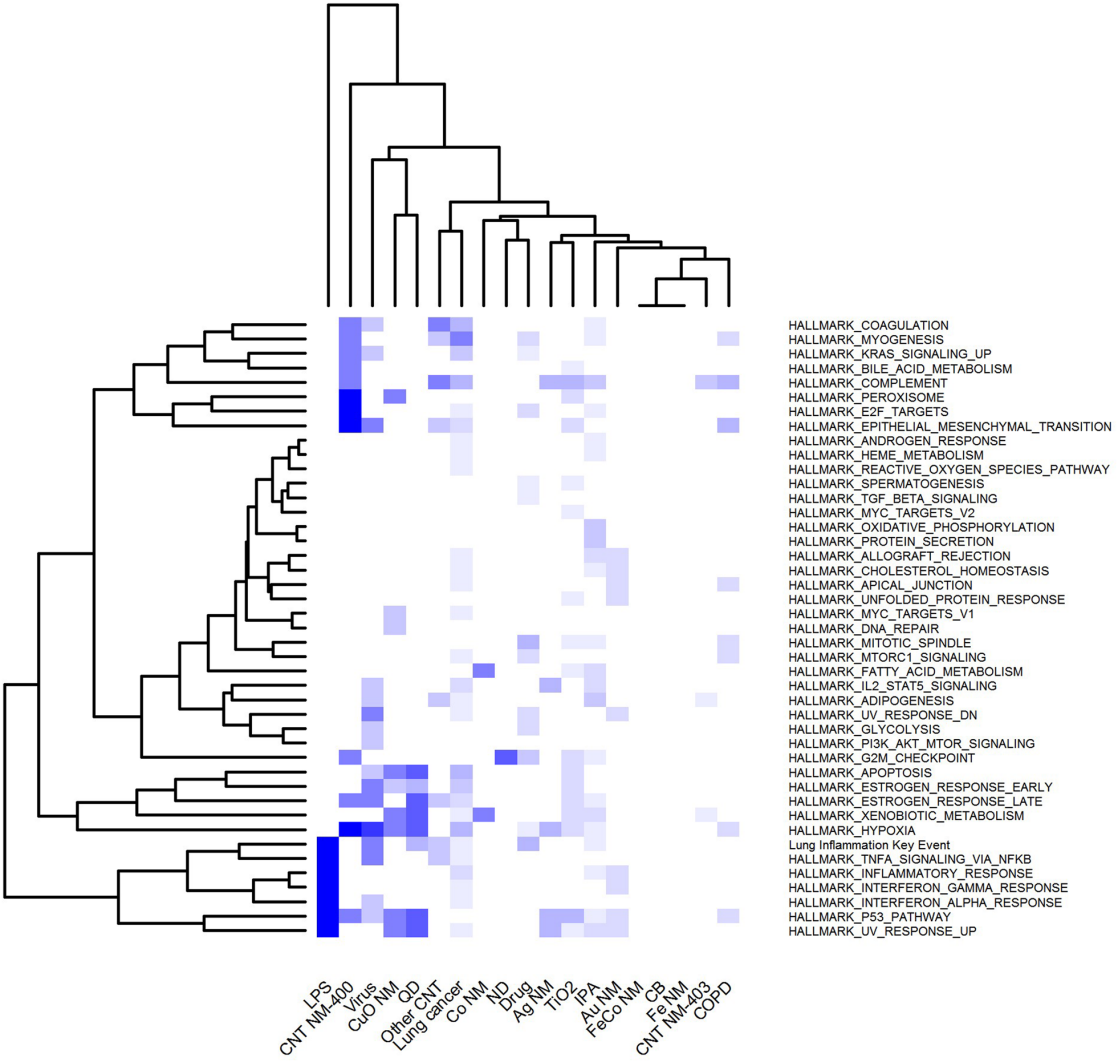
Heatmap comparing HALLMARK pathways across different traits. Each row corresponds to a certain HALLMARK pathway. Different grades of blue indicate the degree to which the corresponding pathway is altered for that trait. Clustering was performed using Euclidean distance and average linkage. The results of the clustering between projects are represented in the column dendrogram; the clustering between HALLMARK pathways across projects is shown in the row dendrogram

Enriched HALLMARK pathways within the different traits were compared in a meta-analysis and results are depicted as a heatmap in Fig. 11. Hierarchical clustering was performed among traits as well as HALLMARK pathways. For this case study we have added a pathway that we created especially for this analysis: the “Lung Inflammation Key Event”. This pathway includes proteins and genes known to be regulated in lungs undergoing inflammation, as collected from 35 papers addressing explicitly this topic. The list of 266 proteins and genes, as well as the citation to the original articles, are included in the Additional file 1: Table S1. The aim of the “Lung Inflammation Key Event” pathway was to gain a comprehensive description of an important key event often present in different AOPs, and particularly in the AOP for lung fibrosis. LPS serves as a positive control for activation of inflammatory response, and it proved to strongly regulate this pathway. Figure 11 shows that different types of NMs exhibit particular behaviors, and the caused alterations resemble distinct traits. This is true also for the “Lung Inflammation Key Event” pathway.

When comparing proteomic signatures, most reliable results are obtained when considering as many projects as possible. PROTEOMAS’ ability to process them in a harmonized and automated manner permits to deal with such a high number of datasets. The ever-increasing number of omic studies being publicly available will allow to develop an increasing understanding of the biological alterations caused by studied traits.

## Discussion

New methodological developments to contribute to the advance of AOPs are crucial in establishing reliable alternative methods for toxicology in line with the 3R-principles of reducing, refining or replacing animal testing. Omic techniques are very promising methods in this regard, precisely due to the potential to provide plenty of information on the MoA of evaluated substances. Currently, most omic-based approaches to unravel toxicity mechanisms rely on transcriptomics [26–29]. Transcriptomics has already proven its potential, e.g. by its contribution to the lung fibrosis AOP [30, 31] and by its involvement in the Genomic Allergen Rapid Detection (GARD) approach for skin sensitization [32, 33].

Proteomics, on the other hand, has the potential to be more descriptive of the adverse outcome, since this method can closer describe the phenotype than transcriptomics. The drawback of proteomics is the heterogeneity among proteomic datasets due to the high variability of methods and instruments used to generate the data. As opposed to microarray- or RNAseq-based transcriptomics, proteomic output does not necessarily contain information on the same set of molecules, i.e. includes a larger number of missing values, which makes comparison of different experiments more challenging.

Here, we present an automated workflow to process proteomic data which allows analysis in a high-throughput manner without subjective bias. Although the workflow can be used to process data from a single dataset as well, its main benefit lies in the possibility of processing a large number of them, for example those found in public repositories. Once a collection of datasets is retrieved from the repository, PROTEOMAS allows in a simple and harmonized way, to process the datasets in a sequential manner.

For each dataset, the workflow identifies a group of proteins that appear altered among evaluated conditions within the dataset, and assigns a series of descriptors, like protein IDs, GO terms, KEGG, HALLMARK and Reactome pathways, among others, altogether highlighting the proteomic signature of each particular dataset, which can be linked to relevant biological changes and by this to phenotypic differences. Such a systematic and harmonized data analysis allows the comparison of results from many different proteomic projects, i.e. by mapping their proteomic signatures. Additionally, it contributes to the reuse of proteomic data, which can be then more easily integrated to the outcome of other omic techniques, like the wealth of transcriptomic and metabolomic results already publicly available.

In the present work, we show that original results from the projects taken from the repository vary only minimally from those originated by PROTEOMAS. In the framework of a case study, we proved the utility of PROTEOMAS for comparing NM-related proteomic data with other lung-related studies. Since this workflow is versatile in processing a large amount of proteomic datasets, we could easily compare the proteomic signature from different NM treatments, to those of other various effectors. A special emphasis was put to inflammation as key event attempting to contribute to the development of AOPs. The same procedure however, could easily be followed to investigate the effect of other substances on other organs. Results can advance directly the development of AOPs and the understanding of the MoA.

In parallel, our workflow aims at facilitating the application of artificial intelligence strategies to describe the effect of evaluated treatments, thus contributing to make proteomic data analysis more FAIR [34, 35]. Simultaneously, our workflow was developed to comply with the DAPRM and DARM of the Omics Reporting Frameworks (TRF and MRF) by the OECD, in order to increase the transparency of proteomic data analysis for regulatory purposes.

## Conclusion

Hazard assessment of chemicals relies mostly on very expensive and time consuming in vivo experiments. The high number of substances which are placed in the market requires the development of alternative methods. However, their adequacy depends on the deep understanding of the substance’s mechanistic effects. Omic studies are extremely useful to provide the required mechanistic knowledge, since they provide a comprehensive description of caused alterations at different molecular levels. However, they are not yet considered as routine methods in regulatory assessments due to the lack of standardization of the computational analysis of the datasets. Workflows for harmonization of the analysis of omic data contribute directly to facilitate the use of omics in regulatory decision making. Most of the efforts in this regard have been made in the field of transcriptomics. Proteomic experiments on the other hand, besides being more descriptive of the phenotype, are not performed in a manner that allows straightforward comparison of results, because the experimental setup and measuring methods do not belong to established platforms, as for transcriptomics. To address this challenge, in this work we introduced a workflow called PROTEOMAS for harmonized proteomic data analysis, precisely intended to facilitate the use of omics in regulatory decision making. Thus the main utility of our workflow is that it can perform meta-analysis of proteomic data from public origin, allowing the comparison of results from different experimental sources, while increasing the transparency of the analysis. Additionally, it is in agreement with Omics Reporting Framework guidelines of the OECD to integrate proteomics to other omic methods used in regulatory toxicology.

In this work to show the robustness and reliability of PROTEOMAS, we run our workflow on 25 different datasets from public origins and obtained comparable results with the source publications. Additionally, we developed a case study, where we performed a meta-analysis to study the toxicological effect of nanomaterials at the lung level, with a particular focus set on inflammation. Altogether, PROTEOMAS is a contribution to the development of alternative test strategies by facilitating the integration of proteomic experiments, while committing to the FAIR principles (Findable, Accessible, Interoperable and Reusable) of computational protocols.

## Methods

### Workflow characteristics

PROTEOMAS is a workflow for efficient processing of MS-based proteomic datasets in a high throughput manner. The workflow is fully automated and implemented in Python (version 3.5) and R (version 4.1.0) in a platform-independent manner (usable under Windows, Linux and MAC). In addition, it can be applied on any dataset, either publicly available or de novo generated by an LFQ approach, which includes multiple replicates for each condition or treatment (n ≥ 3). The corresponding code can be found under https://github.com/AileenBahl/PROTEOMAS.

In brief, PROTEOMAS starts from MaxQuant output files and performs a series of statistical steps, which are explained in more detail in the Results section. The workflow starts with typical data processing steps like filtering, transformation, normalization, imputation and outlier removal. Subsequently, proteins which are significantly altered among conditions are identified. Protein set enrichment analysis is used to identified enriched KEGG, Reactome and HALLMARK pathways as well as GO terms. A flowchart (Fig. 1) summarizing the workflow steps was created using the yEd tool https://www.yworks.com/products/yed.

### Obtaining input data

PROTEOMAS can be used to analyze the user’s own as well as public proteomic datasets. Public datasets may be retrieved from the PRIDE [36] (PRoteomics IDEntifications) Archive, which is a public data repository of MS-based proteomic data (https://www.ebi.ac.uk/pride/archive). The PRIDE Archive includes currently over 20.000 (state November 2022) projects and this number is rapidly increasing. From PRIDE, the user may download raw files for each project of interest and subject them to a MaxQuant analysis. Instead, for many projects MaxQuant output files are available on PRIDE along the corresponding raw data which can be used directly as input for the workflow.

### Raw data analysis

In case only raw data is available for a project of interest, MaxQuant has to be run before PROTEOMAS. MaxQuant [14] is an established proteomics software, which is primarily used for protein identification and quantification, using algorithms specifically developed for the analysis of high-resolution quantitative MS data. It performs data integration and statistical validation for protein inference by using false discovery rates (FDR). MaxQuant output files are tables of the detected peptides, proteins and protein groups. MaxQuant (version 1.6.14) was used in this work to process raw MS-based proteomics files by searching either against human, rat or mouse Uniprot databases (State: March 2021), respectively. The false discovery rate was set to 1% (default value). For advanced protein identification, the ‘Match between runs’ parameter was enabled. Protein normalization and quantification was done in MaxQuant by applying the LFQ parameter, in which the minimum number of unique peptides was set to 1. The workflow’s input is the ‘proteinGroups.txt’ output file generated by MaxQuant analysis, which contains the identified protein groups, all-, razor- and unique peptides, as well as LFQ intensities. Normalized LFQ intensities generated by MaxQuant are exported from the ‘proteinGroups.txt’ file and used for further analysis.

### Statistical analysis

All data cleaning, transformation and filtering steps were performed using basic Python (version 3.8), as well as some standard additional packages like pandas and numpy. In addition, for the statistical analysis some more advanced R packages (R version 4.1.0) were embedded into the Python code using the rpy2 package. For the imputation of values missing completely at random (MCAR) we used the R package missForest [20] with the number of trees set to 30 and the maximum number of iterations set to 3. In addition, the PCAGrid [37, 38] method from the rrcov package is used to automatically detect outlier samples. All arguments were set to default. In case of insufficient metadata, the NbClust [21] package is used for prediction of the optimal number of groups (k) and group assignment using k-means algorithm. Linear modeling was performed using the lm() function from R and false discovery rates (FDRs) are computed using Python’s statsmodels.stats.multitest package. This results in a list of significantly altered proteins for each analyzed dataset with cut-offs set to FDR < 0.05 and log ratio of abundances ≥ log2(1.5).

### Protein set enrichment analysis

Protein set enrichment analysis is a method used for the biological interpretation of the obtained sets of proteins with significantly altered abundances. Different databases or ontologies can be used to this end. In this work, protein enrichment analysis was performed using the R-package ‘fgsea’ [39]. Background sets were obtained from the Human Molecular Signatures Database (MSigDB) [40] (version 2022, human). Uniprot IDs of the analyzed proteomic datasets were mapped to gene names using the Uniprot.ws package from R. Mouse and rat gene names were mapped to human ones using the msigdbr() package. After these id transformations, results from the proteomic experiments are ready to be compared to the background databases.

Different databases are used to obtain information on enriched gene sets. Kyoto Encyclopedia of Genes and Genomes or shortly **KEGG** (www.kegg.jp/kegg/pathway.html) is a bioinformatics database resource for understanding biological and cellular functions as well as biological pathways from a genomic perspective [41]. The database is online available and can be used to analyze and classify genes into their respective functional pathways, which are a collection of reference maps that correspond to a known functional or biological network. The ‘KEGG PATHWAY’ category represents pathway maps in various types of molecular networks, such as reaction and interaction networks for metabolism, cellular processes networks, disrupted reaction and interaction networks of human diseases, as well as chemical structure transformation networks for drug development. Similarly, the **REACTOME** database (https://reactome.org/) [42] contains manually curated pathways describing various molecular processes. For REACTOME gene set in the MSigdb, the original REACTOME pathways have been filtered to remove redundancy between the different sets. The Gene Ontology (**GO**) knowledgebase (www.geneontology.org) describes biological information based on three main layers: biological process (BP), cellular component (CC) and molecular function (MF). **HALLMARK** gene sets represent a collection of well-defined biological states or processes which show coherent expression [40].

In addition to the established databases and gene sets described above, we specifically created the so-called “Lung Inflammation Key Event” gene set. This set incorporates 266 genes that are known to be regulated in lungs undergoing inflammation. These genes were extracted from 35 papers addressing explicitly this topic. The list of included genes, as well as the citation to the original articles, is given in Additional file 1: Table S1. With this gene set we aim at comprehensively describing the important key event of inflammation which is present in many different AOPs, and particularly in the AOP for lung fibrosis.

The Python script created in this work obtains enrichment scores for all of the aforementioned databases. All significantly enriched terms having a FDR less than 0.05 are collected in a single file, which includes the category (e.g. Process, Function, KEGG), the term (e.g. GO identifier), the description, as well as the p-value and the FDR values for each enriched term. The enriched terms generated by PROTEOMAS were used for data interpretation. Within this work, we mainly concentrated on the interpretation of HALLMARK pathways.

### Datasets and application of the workflow

We randomly selected publicly available proteomic datasets from the PRIDE Archive repository, which originate from studies on lung alterations. We focused on pulmonary alterations because we intend to investigate the inhalative toxicological effects of NM in future studies. First, we prioritize cancerogeneous effects induced by NM. Therefore, we compiled a collection of 25 proteomic datasets (Table 1) to generate a preliminary map of lung alterations, eight of which are related to lung cancer and lung cancer treatments. The other projects cover different pulmonary traits as a background set of alteration as well as five studies on NM treatments. All studies were analyzed in an automated manner by the PROTEOMAS workflow. Venn diagrams comparing original findings against those obtained from PROTEOMAS were generated using R’s VennDiagram package.

### Report generation according to requirements of the OECD transcriptomics reporting framework

During data evaluation PROTEOMAS automatically generates a report summarizing relevant information on the data analysis. The recorded information is in line with the requirements laid down in the transcriptomics reporting framework of the OECD. The requirements are summarized in Table 3.

**Table 3:** Selection of requirements of the OECD Transcriptomics Reporting Framework which are relevant for PROTEOMAS

Task	Required information
Normalization	- Normalization method - Background data subtraction - Method of background calculation - Weighting procedure - Log transformation - Data trimmed? - Control samples removed before normalization? - Formulas - Link/repository/accession number for deposited normalized data + format + description of raw data tables
Data filtering	- Low signal intensities - High variability between technical replicates - Which methods? - Which cut-offs?
Outlier removal	- Method for identification and thresholds - Exclusion at which processing step - List of samples excluded and per sample Justification - Removal before or after normalization and Justification
Discovery of differentially abundant molecules (DAMs)	- Name and version of software - Operating system - Name and version of additional libraries - Availability of software, hyperlinks or source codes - Table of all contrasts / conditions compared for DAM identification - Table of number of samples in each group for DAM identification - Identification of samples with expected covariances (due to shared conditions during processing) - Identification of technical replicates - Name and description of statistical approach - Data transformation performed - For effects models: Specification of effects models used and effects that were modelled - For pairwise comparison approaches: specification of test and values (any transformation or adjustment) being used - Specification of decision criteria (nominal alpha value, p-value threshold, multiple testing correction method, adjusted threshold value, log fold-change cut-off level) including exact order of operations - Output and supporting files according to the file manifest, list all files including a description, describe rows and columns of tables, analysis scripts, software configurations or tables of metadata

## Supplementary Information

**Additional file 1. Supplemental Table: **Proteins and genes known to be regulated in lungs under inflammation conditions. It was built from 35 publications addressing explicitly this subject. The list includes 266 proteins and genes, as well as the citation to the original articles.

## Data Availability

ALL raw data can be found on the PRIDE archive under the following accessions: PXD007223 [23], PXD000861 [43], PXD018895 [44], PXD000853 [22], PXD005698 [45], PXD005733 [46], PXD007137 [47], PXD004818 [48], PXD007180 [49, 50], PXD020470 [51], PXD021685 [52], PXD007148 [50], PXD016664, PXD014022 [24], PXD005834 [53], PXD018569 [54], PXD023041 [55], PXD013244 [56], PXD016148 [57], PXD019267 [58], PXD018900 [59], PXD005970 [60], PXD025423 [61]. To support FAIR principles, the processed datasets and results from downstream analyses can be accessed via https://github.com/AileenBahl/PROTEOMAS/AnalysisResults. All PROTEOMAS scripts can be found on GitHub under the following link: https://github.com/AileenBahl/PROTEOMAS.

## Abbreviations

AOP: Adverse outcome pathways
DAM: Discovery of differentially abundant molecules
DAPRM: Data acquisition and processing reporting module
DARM: Data analysis reporting module
DDA: Data-dependent acquisition
FAIR: Findable, accessible, interoperable and reusable
FDR: False discovery rate
GARD: Genomic allergen rapid detection
GSEA: Gene set enrichment analysis
kNN: K-nearest neighbors
LFQ: Label-free quantification
LOD: Limit of detection
MCAR: Missing completely at random
MoA: Mode-of-action
MRF: Metabolomic reporting frameworks
MS: Mass spectrometry
OECD: Organisation for Economic Cooperation and Development
TRF: Transcriptomic reporting frameworks
